# Unraveling the Tapestry of Pain: A Comprehensive Review of Ethnic Variations, Cultural Influences, and Physiological Mechanisms in Pain Management and Perception

**DOI:** 10.7759/cureus.60692

**Published:** 2024-05-20

**Authors:** Neelay Shah, Rida Qazi, Xiang-Ping Chu

**Affiliations:** 1 Neurology, University of Missouri Kansas City School of Medicine, Kansas City, USA; 2 Biomedical Sciences, University of Missouri Kansas City School of Medicine, Kansas City, USA

**Keywords:** neuropathic pain treatment, integrative pain management strategies, pharmacological approaches to pain relief, multidisciplinary pain management, pain assessment tools, psychological aspects of pain management, non-pharmacological pain interventions, opioid use in pain management

## Abstract

The medical management of pain is a nuanced challenge influenced by sociocultural, demographic, and ethical factors. This review explores the intricate interplay of these dimensions in shaping pain perception and treatment outcomes. Sociocultural elements, encompassing cultural beliefs, language, societal norms, and healing practices, significantly impact individuals' pain experiences across societies. Gender expectations further shape these experiences, influencing reporting and responses.

Patient implications highlight age-related and socioeconomic disparities in pain experiences, particularly among the elderly, with challenges in managing chronic pain and socioeconomic factors affecting access to care.

Healthcare provider attitudes and biases contribute to disparities in pain management across racial and ethnic groups. Ethical considerations, especially in opioid use, raise concerns about subjective judgments and potential misuse. The evolving landscape of placebo trials adds complexity, emphasizing the importance of understanding psychological and cultural factors.

In conclusion, evidence-based guidelines, multidisciplinary approaches, and tailored interventions are crucial for effective pain management. By acknowledging diverse influences on pain experiences, clinicians can provide personalized care, dismantle systemic barriers, and contribute to closing knowledge gaps, impacting individual and public health, well-being, and overall quality of life.

## Introduction and background

The classification and study of race have long been subjects of extensive scientific investigation and societal discussions throughout history [1]. Human populations exhibit variations in physical characteristics, genetics, and geographic origins, which form the basis for understanding race [2]. However, scientific advancements have challenged the simplistic notion of race as a fixed and distinct category, highlighting the incredibly complex nature of human diversity [3].

Through genetic studies, we have gained valuable insights into human populations and their genetic variations [3, 4]. The analysis of genomic data has illuminated the intricate patterns of historical human migrations and intermixing [4].

Extensive research has also been conducted on the impact of race on individuals' lived experiences and health outcomes [4]. Disparities in health among different racial groups, especially in the United States, have been well-documented [5]. A recent study published in the American Journal of Public Health in 2020 investigated health disparities between racial minorities and white Americans [1]. The findings revealed that racial minorities such as African Americans and Hispanic Americans experience higher rates of chronic diseases, limited access to healthcare services, and increased mortality rates compared to their white counterparts [1, 4]. These findings underscore how race significantly influences various aspects of individuals' lives. Therefore, it is crucial to address systemic inequities and promote racial justice [1]. Furthermore, the impact of race on people's health and lived experiences has been scientifically proven, necessitating efforts to address disparities and advocate for equality [1, 2]. Understanding the intricate nature of race is crucial for fostering inclusivity and working towards a society that values diversity while upholding principles of fairness and justice [6].

## Review

Classification of pain

Pain can manifest itself in various ways, with its classification often based on different factors such as the underlying cause, duration, and location [7]. Having a comprehensive understanding of the various types of pain is crucial for accurate diagnosis and implementing effective strategies for pain management. The following are some common forms of pain:

Chronic pain lasts beyond the typical healing period and persists for more than three to six months [8]. It can be attributed to an underlying medical condition like arthritis, fibromyalgia, or neuropathy [9] or it may occur without a clear cause [10]. Managing chronic pain can be intricate and challenging as it often impacts an individual's quality of life along with their functioning and emotional well-being [9, 10].

Pain associated with cancer can arise from the tumour itself, various treatments including surgery, radiation, and chemotherapy, or other factors related to cancer [9]. This pain can be categorized as acute or chronic, nociceptive or neuropathic, and its intensity and location may vary depending on the specific type of cancer and its stage [9]. Adequate management of cancer-related pain is crucial for enhancing the quality of life for individuals affected by this disease [9].

Acute pain typically arises as a temporary warning sign of injury or illness. It is commonly triggered by specific events or conditions such as surgery, trauma, or infection [10]. Acute pain usually has well-defined characteristics in terms of its onset, intensity, and location; moreover, it is expected to improve once the underlying cause is treated [10].

Psychogenic pain refers to pain that is influenced or worsened by psychological factors like stress, anxiety, or depression [11]. Although there may not be a clear organic cause for this type of pain, it can be severe and greatly impact the patient's daily life [11]. Psychological approaches such as cognitive-behavioural therapy are often employed in managing psychogenic pain [11].

Nociceptive pain occurs when specific nerve endings called nociceptors are activated in response to tissue damage or inflammation [11]. This type of pain is usually localized and can be described as aching, throbbing, or sharp in nature [10]. Examples of nociceptive pain include pain from a strained muscle, arthritis in the joints, or a surface cut [11].

Radicular pain arises from irritation or compression of a nerve root in the spinal cord, often caused by conditions like herniated discs or spinal stenosis [12]. It can cause sharp and shooting pain that travels along the nerve pathway and may be accompanied by numbness, tingling, or weakness in muscles [13].

Neuropathic pain arises from damage or dysfunction of the nervous system [14]. It can be caused by conditions such as advanced diabetes, uncontrolled autoimmune diseases, various vasculitides, post-herpetic neuralgia, and mechanical compression [6, 12, 15]. Neuropathic pain is characterized by unusual sensations like shooting or burning pain, tingling, numbness, or heightened sensitivity [16]. It often persists for a long time and can pose challenges for effective treatment [17].

Intra-group variations in pain perception and reasoning among people of the same ethnic or racial background are referred to as intra-ethnic differences. These differences encompass the diverse experiences of pain and the cognitive processes involved in how individuals perceive and interpret pain [15, 18]. It's crucial to recognize that even within these groups, there is significant diversity, despite shared cultural and genetic factors [14]. To fully understand intra-ethnic differences in pain perception and reasoning, several key factors need to be considered.

Cultural influences have a profound impact on how individuals within an ethnic group perceive and reason about pain [18]. Cultural beliefs, values, and practices related to pain, as well as the social and familial context in which pain is experienced, all shape individual experiences and subsequent responses [19]. These cultural factors exhibit considerable variability among individuals with the same ethnic background, influencing how they interpret, communicate about, and manage their pain [20].

Psychosocial factors also contribute to the observed intra-ethnic differences in pain experiences and reasoning [19]. Individual beliefs, attitudes, and previous experiences with pain play crucial roles in how individuals perceive and interpret their pain [21]. Various social and psychological dynamics can lead to significant variations in pain perception and reasoning among individuals within the same ethnic group, as stated in Innes [22]. Differences in pain experiences and reasoning within ethnic groups can also be attributed to health disparities [23]. Factors like healthcare accessibility, quality of care, and socioeconomic status impact how people encounter pain and their ability to seek appropriate treatment [24]. These disparities are evident even among individuals from the same ethnic background when it comes to pain management approaches and outcomes [25]. Considering genetic and biological factors is crucial as well [13]. While there may be shared genetic factors within an ethnic group, individual genetic variations contribute to differences in how people perceive and process pain [26]. Additionally, biological elements such as neurotransmitter function, hormonal influences, and ion channel activity also affect one's experience of pain [27].

Environmental influences encompass various aspects like socioeconomic status, educational attainment, and exposure to different stressors. These factors play a significant role in explaining intra-ethnic differences in pain perception and reasoning [28]. They shape how individuals within a specific ethnic or racial group experience pain and cope with it [20]. In conclusion, discussions regarding intra-ethnic differences in pain perception and reasoning require a meticulous and nuanced approach [29]. Understanding the complicated aspects of these variations, which encompass cultural, psychological, health-related, genetic, and environmental influences [30, 31], is crucial for comprehending how individuals perceive pain and enhancing personalized pain management techniques [20]. Continuous research efforts aim to unravel the intricate interactions between these factors in diverse ethnic and racial populations, with the goal of advancing our comprehension of pain experiences and optimizing pain treatment [32].

Potential mechanisms of pain

Varying Types of Ion Channels and Associated Interference in Pain

Voltage-gated ion channels play a crucial role in the creation and transmission of electrical signals in excitable cells, especially neurons [33]. They are activated by changes in the membrane's electrical potential and help facilitate the movement of ions, such as sodium, potassium, and calcium, across the cell's outer layer. This activity is vital for transmitting nerve impulses and signalling [33].

Sodium Channels

Voltage-gated sodium channels, such as Nav1.7, Nav1.8, and Nav1.9, are primarily found in sensory neurons involved in perceiving pain [34]. These channels play a significant role in initiating and spreading action potentials, which help transmit pain signals from peripheral tissues to the central nervous system [34].

Nav1.7 (SCN9A): Nav1.7, also known as SCN9A, is a voltage-gated sodium channel predominantly found in sensory neurons responsible for the perception of pain [11]. This sodium channel is of paramount importance in the field of pain research due to its central role in nociception. Mutations in the SCN9A gene that encodes Nav1.7 have been extensively associated with various pain disorders, emphasizing its critical role in pain perception and management [35]. These genetic variations can lead to either a loss of function, resulting in congenital insensitivity to pain, or a gain of function, causing inherited erythromelalgia or paroxysmal extreme pain disorder.

Nav1.7 is crucial for initiating and propagating action potentials within sensory neurons, making it a key player in transmitting pain signals from peripheral tissues to the central nervous system [36]. Its function is highly specialized, allowing it to sense and transmit pain signals with remarkable precision. The mechanism by which it accomplishes this involves a fast and selective sodium ion conductance, which contributes to the depolarization of the neuron's membrane, ultimately leading to the firing of action potentials.

Understanding Nav1.7's functions and genetic associations is not only essential for basic neuroscience research but also holds promise for the development of potential therapeutic approaches to manage pain effectively. Researchers and pharmaceutical companies have been actively exploring drugs that target these sodium channels, including Nav1.7, as potential treatments for various chronic pain conditions [37]. The hope is that by modulating the activity of Nav1.7, it may be possible to provide relief to those who suffer from debilitating chronic pain, thereby significantly improving their quality of life [38].

Nav1.8 (SCN10A): Nav1.8, coded by the SCN10A gene, is another integral sodium channel in sensory neurons, primarily responsible for transmitting pain signals triggered by mechanical and thermal stimuli [38]. Like Nav1.7, Nav1.8 plays a vital role in nociception, expanding our understanding of the complexity of the pain signalling pathway. It is essential for generating action potentials in peripheral nerve endings, which are crucial for relaying pain information to the central nervous system [39].

Nav1.8's involvement in pain signalling is multifaceted. It possesses unique biophysical properties that allow it to activate and inactivate at relatively depolarized membrane potentials, making it well-suited to detecting and transmitting pain signals triggered by mechanical and thermal stimuli [38]. This specialization underscores its significance in the transmission of specific types of pain [38].

Similar to Nav1.7, Nav1.8 is of great interest to researchers and pharmaceutical companies as a potential therapeutic target for managing chronic pain conditions [39]. Investigating this sodium channel offers promising avenues for the development of pain management drugs that can selectively modulate its function, potentially offering relief to individuals suffering from chronic pain [39].

Nav1.9 (SCN11A): Nav1.9, coded by the SCN11A gene, is another sodium channel present in sensory neurons, specifically those associated with pain perception [14]. It has been linked to inflammatory pain and is believed to contribute to the generation of persistent or chronic pain signals [40], highlighting its role in chronic pain conditions. Nav1.9 contributes to the excitability of sensory neurons, facilitating the transmission of pain information [41]. Its properties make it well-suited to detect and transmit pain signals associated with inflammation and other chronic pain states.

The involvement of Nav1.9 in chronic pain mechanisms provides a valuable avenue for research and therapeutic development. This understanding holds promise for potential therapeutic interventions in chronic pain management. Just like Nav1.7 and Nav1.8, Nav1.9 is under scrutiny for its potential as a target for developing drugs to treat chronic pain, and it is a subject of active research by both scientists and pharmaceutical companies. By focusing on the modulation of Nav1.9 function, researchers aim to develop treatments that can effectively manage chronic pain conditions, potentially offering relief to individuals who have long endured such pain.

In summary, Nav1.7, Nav1.8, and Nav1.9 are integral components of the pain signalling pathway, and understanding their roles and genetic associations has significant implications for pain management and potential treatments for various chronic pain conditions. These sodium channels provide critical insights into the intricate mechanisms of pain perception, and ongoing research endeavours aim to harness this knowledge for the benefit of individuals experiencing chronic pain.

Potassium Channels

Potassium channels, such as Kv1.1 and Kv1.2, are crucial in regulating the excitability of neurons [42]. They play a role in controlling the duration and repolarization of action potentials, and when specific potassium channels malfunction, it can lead to hyperexcitability and pain-related conditions [43, 44].

Kv1.1: Kv1.1 is a specific type of voltage-gated potassium channel that serves as a crucial regulator of neuronal excitability [45]. Its significance lies in its ability to influence how neurons fire and to ensure effective communication between nerve cells. Disruptions or mutations in Kv1.1 channels have been associated with several neurological disorders, including episodic ataxia and myokymia. These conditions can involve symptoms related to pain, underscoring the channel's pivotal role in pain modulation [46].

The study of Kv1.1 opens up avenues for understanding the intricate mechanisms that underlie neuronal excitability. Researchers are investigating the potential therapeutic applications of targeting Kv1.1 channels to manage disorders associated with hyperexcitability and offer relief from the pain symptoms that often accompany them. This exploration holds promise for both fundamental neuroscience and clinical medicine, providing a deeper understanding of the role potassium channels play in the nervous system.

Kv1.2: Kv1.2, another voltage-gated potassium channel, plays a vital role in shaping neuronal action potentials [47]. It is distributed widely throughout the nervous system, and found in various types of neurons. Dysregulation of Kv1.2 channels has been associated with conditions such as epilepsy, where excessive neuronal excitability can lead to seizures and related experiences of pain [48].

Understanding Kv1.2 and its role in neuronal function offers insight into the mechanisms that govern the excitability of neurons in various parts of the nervous system. This knowledge not only advances our understanding of fundamental neuroscience but also carries practical implications for clinical medicine. Researchers are exploring the potential to target Kv1.2 channels as a means of managing disorders characterized by hyperexcitability and the accompanying experiences of pain [48].

Recent research endeavours have focused on unravelling the intricate mechanisms through which potassium channels, like Kv1.1 and Kv1.2, impact neuronal excitability [48]. These studies aim to explore potential therapeutic applications by targeting these channels to manage disorders associated with hyperexcitability, offering a path to alleviate pain syndromes.

Calcium Channels

Voltage-gated calcium channels, such as Cav2.2 and Cav3.2, play a pivotal role in the regulation of neurotransmitter release and can profoundly impact pain signalling [49]. These channels influence pain perception and transmission by affecting the excitability of pain-sensing neurons, revealing their critical role in nociception.

Cav2.2: Cav2.2, also known as N-type calcium channels, holds particular relevance in the context of pain perception [50]. These channels are present in both central and peripheral neurons, and they play a fundamental role in regulating neurotransmitter release, including substances involved in pain signalling, such as substance P. Researchers have actively investigated targeting Cav2.2 channels as a potential approach to managing pain, especially chronic pain conditions. By modulating the activity of these channels, researchers aim to regulate pain signalling, providing relief to those experiencing chronic pain [9].

Cav3.2: Cav3.2, often referred to as T-type calcium channels, represents another type of voltage-gated calcium channel that contributes significantly to pain signalling [51]. These channels are involved in generating burst firing patterns in sensory neurons, impacting the transmission of pain signals. Dysregulation of Cav3.2 channels has been linked to various pain disorders, making them a focal point for research in the field of pain management [9].

Recent research has provided valuable insights into the specific functions of calcium channels within pain pathways. Scientists have been actively working on developing selective regulators and inhibitors for these channels to influence the perception and transmission of pain. The ongoing investigation into the role of calcium channels in relation to pain holds great promise for gaining a deeper understanding of pain-related conditions and finding more effective ways to manage them. The targeted modulation of calcium channels presents a potential avenue for the development of innovative pain management strategies, offering hope to individuals suffering from various pain disorders.

TRP Channels

Transient receptor potential (TRP) channels form a diverse group of ion channels that play important roles in various sensory processes, including pain [52]. These TRP channels, such as TRPV1 (known as the vanilloid receptor), TRPM8 (referred to as the cold receptor), and TRPA1 (recognized as the chemical irritant receptor), are expressed in sensory neurons and can be activated by different stimuli. As a result, they have an impact on how we perceive pain and respond to thermal or chemical stimuli [53, 54].

TRPV1: Also known as the vanilloid receptor, TRPV1 is well known for its involvement in our perception of pain and sensitivity to heat and capsaicin - the compound responsible for making chili peppers spicy [55]. It is primarily found in sensory nerve endings where it plays a crucial role in transmitting information about potentially harmful levels of heat or irritating chemicals. This receptor contributes significantly to our experience of pain and discomfort, acting as a sentinel for warning us of potential threats to our well-being. Moreover, beyond its role in pain perception, TRPV1 also plays a part in regulating body temperature and contributing to the inflammatory response.

TRPM8: Often referred to as the cold receptor, TRPM8 responds specifically to cold temperatures and cooling agents like menthol [56]. Activation of the TRPM8 receptor can result in a cooling sensation, which has implications not only for sensory experiences but also for relieving pain in conditions such as neuropathic pain. Researchers are keen on exploring the therapeutic potential of targeting TRPM8 to mitigate pain, especially in situations where cooling sensations can provide relief from discomfort or even contribute to innovative pain management strategies [54].

TRPA1: Known as the chemical irritant receptor, TRPA1 can be activated by various chemical irritants found in environmental pollutants and spicy foods. This receptor is involved in both pain perception and inflammatory processes, making it a significant player in the body's defence mechanisms against potential harm. Understanding TRPA1's role is instrumental in comprehending how the body senses and responds to irritants and painful stimuli, which can aid in developing strategies for pain management, particularly in the context of inflammatory pain.

The complex interplay of these transient receptor potential (TRP) channels, like TRPV1, TRPM8, and TRPA1, in sensory processing and pain perception has captured the attention of researchers. Recent scientific investigations have shed light on how these channels contribute to the sensory experience of pain and discomfort. Moreover, the ability to target and modulate these channels presents a promising avenue for innovative pain management strategies.

Genetic Variations Influencing Ion Channel Activity

The role of ion channels in pain sensitivity has been a topic of significant scientific interest. These channels play a critical role in transmitting and modulating pain signals within the intricate network of the nervous system. Genetic variations or mutations in ion channel genes can alter their function, potentially affecting one's perception and sensitivity to pain.

One specific example that has garnered substantial attention is the Nav1.7 voltage-gated sodium channel, encoded by the SCN9A gene [57]. Mutations in SCN9A have been associated with various pain disorders, including inherited erythromelalgia and paroxysmal extreme pain disorder [58, 59]. These mutations result in hyperexcitability in nociceptive neurons, leading to heightened sensitivity to pain and the occurrence of spontaneous pain experiences [60].

Similar findings have been observed with mutations found in other ion channel genes like Nav1.8 (encoded by SCN10A) and Nav1.9 (encoded by SCN11A), which are linked to altered perception of pain [61, 62]. These channels are expressed in sensory neurons and contribute to the generation and transmission of action potentials [62]. Variations within these channels can influence the excitability and firing characteristics of pain-sensing neurons, thereby impacting pain thresholds and responses to pain stimuli [63].

In addition to voltage-gated sodium channels, ion channels involved in neurotransmitter release and synaptic transmission, such as calcium channels and potassium channels, also play a significant role in the processing of pain signals [14]. Genetic variations within these channels may potentially affect neurotransmitter release, neuronal excitability, and the balance between excitatory and inhibitory signalling mechanisms, ultimately influencing an individual's sensitivity to pain [28].

Moreover, ion channels that play a role in inflammatory processes, such as transient receptor potential (TRP) channels, have been implicated in how pain is regulated [64]. TRP channels like TRPV1, TRPA1, and TRPM8 contribute to the transmission of pain signals and respond to various stimuli [65]. Genetic variations within these TRP channels can potentially affect their sensitivity to pain-inducing stimuli, thereby leading to altered pain responses [66].

Modulating Ion Channels for Future Applications

Overall, the presence of variations within ion channels can greatly influence pain sensitivity and how pain is perceived [30]. It is crucial to gain a comprehensive understanding of these variations and their functional implications in order to unravel the complex molecular mechanisms underlying pain disorders. This understanding will also aid in developing targeted therapeutic approaches for effective pain management [61]. Further research should be conducted to uncover the precise roles played by specific ion channels in the processing of pain and explore potential therapeutic interventions based on these findings [67]. This ongoing research has the potential to transform our approach to pain management, improving the quality of life for individuals affected by various pain conditions.

Sociocultural factors affecting pain perception

Sociocultural differences have a significant impact on how individuals perceive pain [68]. Cultural beliefs regarding the nature and significance of pain vary across societies, shaping people's expectations and responses when it comes to dealing with painful sensations [1].

For example, different societies may have varying perspectives on pain, seeing it either as a natural part of life or as an indicator of strength, whereas others may perceive it as a sign of vulnerability or cause for worry [69]. These cultural beliefs greatly influence how individuals perceive and experience pain.

Additionally, language itself plays a significant role in shaping our understanding and perception of pain [70]. Various cultures have their own unique vocabularies and ways of describing sensations related to pain [24]. Different languages have extensive and intricate words to express pain. [71]. These linguistic variations can impact how individuals interpret and communicate their experiences with pain, ultimately shaping their perception [69].

Moreover, societal norms and expectations regarding the expression of pain can also shape how individuals perceive it [72]. In certain Western cultures, openly expressing pain or seeking medical attention might be encouraged and socially acceptable [73]. Conversely, other cultures may discourage overt displays of pain and instead emphasize stoicism or self-reliance [14]. These social norms surrounding the expression of pain significantly influence individuals' perception of it and their willingness to seek help or share their experiences with others [58].

Finally, cultural healing practices also contribute to the way we perceive pain [9]. Various traditional healing practices, such as acupuncture, herbal remedies, and spiritual rituals, have an impact on how people perceive and manage pain [74]. These cultural practices can shape individuals' beliefs about finding relief from pain and also influence their pain tolerance levels [75]. The availability and preference for different types of pain management methods can vary across cultures, resulting in diverse perceptions of pain and responses to it [20].

In addition to cultural influences, societal roles and gender expectations can further shape the way people experience pain [76]. Research suggests that societal expectations related to masculinity and femininity can affect how individuals report and respond to pain [14]. For instance, some Eastern cultures may expect men to show more stoicism and be less likely to express their discomfort than women compared to Western norms [77]. These gender-based expectations play a role in how people perceive and communicate their pain experiences, contributing to sociocultural variations in pain perception [64].

It is important to note that these sociocultural differences are not universally applicable, as there are individual variations within cultures as well [78]. Furthermore, the perception of pain is a complex phenomenon influenced by various factors, including biology, psychology, and social dynamics. Recognizing the sociocultural differences in how people experience pain can assist healthcare providers in delivering culturally sensitive care while tailoring effective strategies for managing pain based on the diverse needs of individuals [79].

Patient implications

Age

The medical field has not thoroughly explored the topic of pain, especially in pediatric cases. In a study conducted in 2020, data from 636 girls under the age of 18 diagnosed with amplified pain syndrome revealed different experiences based on whether they had diffuse or localized pain symptoms [80]. Those with diffuse pain reported higher levels of intensity, mental health challenges, and poorer functioning compared to their peers. On the other hand, those with localized pain more consistently mentioned a specific trigger event for their symptoms, although divorced parents were found to have a stronger association with diffuse pain [79]. Additionally, individuals who experienced a constant duration of pain rather than intermittent flares also reported higher intensity and lower functioning. Amplified pain syndrome in adolescent girls can exhibit a wide range of manifestations [80].

While conditions like delirium, pressure ulcers, falls, and incontinence are commonly recognized as geriatric syndromes, it is crucial to acknowledge that chronic pain is prevalent among older patients and is often linked to various age-related syndromes as well. In the year 2018, more than half (55.2%) of participants aged 65 or older reported experiencing chronic pain that lasted for at least three months [80]. Among those with chronic pain, a significant majority (58.6%) rated their symptoms as severe or unbearable. Surprisingly, only 41.4% of these patients received treatment with analgesics for their pain symptoms [81]. The study also found strong correlations between chronic pain and functional limitations, falls, cognitive issues, and mood disturbances [82]. It's important to note that using various analgesic medications like opioids and NSAIDs can increase the risk of delirium, which poses challenges in managing chronic pain in elderly patients who are already at higher risk. However, addressing and treating pain is crucial for maintaining the quality of life of these patients, as it has been linked to delirium as well [82].

Socioeconomic Factors

A recent survey conducted in 2021 among individuals diagnosed with complex regional pain syndrome explored the impact of severe pain symptoms on economic behaviour. Among the 251 patients surveyed from 37 hospital institutions, a significant number (206 individuals) reported economic disruptions due to their deteriorating physical condition [83]. Interestingly, wealthier patients consistently had better access to early care during the course of their disease progression. This gap exists because these individuals have a strong understanding of health and are able to take time off work and afford expensive medical services. Many wealthier patients have private health insurance, so they don't experience long wait times for their care [67]. This becomes a problem when it comes to syndromic pain because the prolonged pain caused by delayed diagnosis changes how the body reacts to pain [19, 57]. As a result, the pain can become more intense [57].

Gender Biases

Studies consistently show that women are more likely than men to experience, talk about, and seek treatment for chronic pain [50]. This difference can be partly explained by societal expectations of masculinity that associate pain with weakness [50]. Consequently, many men worry that admitting their pain will make them appear incapable of handling their responsibilities or burdensome to others. In addition, women generally have lower thresholds for tolerance of pain, which makes them more prone to experiencing severe and unpleasant pain [50]. This is especially true for middle-aged women who are primarily affected by autoimmune conditions that cause joint and muscle pain as the main symptoms [50]. While the exact reasons for the differences in how men and women perceive pain are not completely understood, there is evidence to suggest that female sex hormones like estrogen and genetic factors, including variations in pain-related genes specific to each gender, may have a role to play [50].

Factors Related to Healthcare Providers

In 2023, a comprehensive review of 27 studies involving 599 healthcare providers was conducted to investigate the attitudes of providers towards managing chronic noncancer pain [14, 84]. The findings indicated that providers generally felt more comfortable and willing to prescribe medication with addictive properties when patients were actively involved in self-managing their pain. This was especially true when clear prescribing policies and drug monitoring programs were in place, long-term provider-patient relationships existed, and interprofessional support was readily accessible [9, 85]. Factors that decreased provider comfort included uncertainty about the effectiveness of certain treatments, concerns for patient well-being, past negative experiences with specific patients, challenges in implementing treatment guidelines, as well as organizational barriers such as limited appointment time and excessive documentation requirements [32, 86].

Many of these disparities regarding the medical treatment of pain among different racial backgrounds can be attributed to conscious or unconscious biases held by certain healthcare providers. These biases are often based on unfounded beliefs about minority populations [24]. In a primary healthcare setting, both Black and Hispanic American patients are often considered more likely to undergo additional screening for potential drug abuse compared to White individuals [58]. In certain situations, the race of the patient can influence opioid prescription patterns, depending on the gender of the physician and their cognitive load at that time [1]. Specifically, male physicians were found to be less inclined to prescribe opioids to Black patients when their cognitive load was lower. Conversely, they were more likely to do so when their cognitive load was higher [87].

Ethics regarding pain management

The medical management of pain has become a particularly controversial topic from an ethical perspective as clinicians must largely rely on a subjective scale to form their clinical judgements. The threshold of pain two individuals can withstand, even with the same ailment, may differ substantially. In an analysis involving 13,765 patients with chronic pain, it was found that a total of 77% of patients displayed at least one anomalous biomarker [37]. Additionally, powerful agents like opioids that are used to manage pain come with a plethora of risks and carry a highly addictive profile. As opioids are metabolized via P450 enzymes within the liver and cleared via renal elimination, their dosage must be appropriately adjusted in patients with hepatic and/or renal dysfunction to avoid potentially fatal toxicity. 

However, in certain instances including terminal illness and acute postoperative or traumatic pain, the therapeutic benefits of opioids outweigh the myriad of associated risks. Similar to other drugs, prolonged use leads to the development of tolerance, necessitating increased and more frequent doses in order to maintain the same effect. This phenomenon gives rise to a pattern of heightened consumption which can lead towards patients developing opioid-induced hyperalgesia, where they paradoxically experience heightened sensitivity to pain as a result of the medication [88]. Therefore, in cases where short-term opioid use is warranted, it becomes crucial to vigilantly monitor reported pain levels and objective pain indicators, such as responses to stimuli and facial expressions.

In response to the opioid crisis, physicians have had to reflect and restructure the role that they played in the misuse and subsequent addiction that overcame countless patients. These victims of the crisis placed trust in physicians as pillars of objective data and scientific knowledge, and as a result, faced extensive damage in infinite domains of their lives. In an analysis spanning between 2014 and 2016, which involved a survey of 1,588 patients receiving chronic opioid therapy, it was observed that 86.3% of patients within control groups who were not exposed to opioid risk reduction initiatives endorsed trust in physician judgment as compared to 77.9% of patients who were exposed to such initiatives [89]. This marginal discrepancy between control and test groups suggests that it may be possible to implement opioid risk reduction initiatives while upholding high levels of trust between physicians and chronic opioid therapy patients. 

Opioids are not indicated in the management of chronic pain, and instead, efforts are focused on addressing the underlying cause of pain. Still, there are numerous “wear and tear” conditions including osteoarthritis that most individuals will develop with advancing age regardless of the lifestyle modifications that they implement to reduce their risk. Among the elderly population, chronic pain is linked with substantial suffering, disability, heightened social isolation, greater costs and burdens to the healthcare system, and even increased mortality. The prevalence of chronic pain is said to peak at the age of 60; however, the plateau in prevalence observed in the following years has been attributed to a 57% elevated susceptibility to mortality among elderly individuals grappling with chronic pain [21]. The deeply detrimental effects of chronic pain in the elderly underscore the critical importance of mastering this transition of the medical management of acute to chronic pain. 

Research of pain involving placebo trials

Placebo controls play a crucial role in the realm of pain research involving placebo trials [90]. These controls serve as an indispensable tool in conducting randomized controlled trials (RCTs), providing a baseline to determine whether a treatment outperforms the placebo or proves ineffective [88]. In the context of chronic pain, where conscious expectation has proven unreliable in predicting placebo effects, the need for rigorous placebo-controlled trials becomes even more evident [91].

Addressing this observed phenomenon, Harvard professor Ted Kaptuck delved into the realm of uncertainty and deception, aiming to unravel their necessity in eliciting placebo effects [90]. Double-blind RCTs, widely considered the gold standard, introduce uncertainty by informing the patient, "You may receive the drug or placebo" [91]. Kaptuck's research yielded fascinating insights, revealing that the mere prospect of receiving the drug led to increased positive responses among pain patients [92]. In fact, placebo responses made up a substantial portion, ranging between 50% and 75% of drug responses [90].

Kaptuck further explored deceptive placebo experiments, where patients were falsely informed that they would receive a powerful drug [89]. Interestingly, the research indicated that a greater likelihood of receiving medication led to significantly lower placebo responses [93]. The heightened expectation of pain relief among patients bred greater dissonance, ultimately resulting in weaker placebo responses [94].

In contrast, open-label placebo trials took a different approach by openly informing patients, "I'm prescribing you a placebo" [87]. Surprisingly, this approach resulted in a remarkable improvement in pain symptoms, commonly exceeding 50% compared to patients in 'no additional treatment' controls [86]. In summary, Kaptuck's research suggests that, while deception proved unsatisfactory, uncertainty, as seen in double-blind RCTs, performed similarly to open-label trials [92]. This intriguing finding implies that neither uncertainty nor deception may be essential for eliciting placebo effects [95].

Differences in treating pain

Pain treatment can vary significantly across different racial and ethnic groups due to a combination of various factors [96]. One important factor is how individuals from different backgrounds respond to medication. Genetic variations can lead to differences in how pain medications are metabolized by people from diverse backgrounds [97]. For instance, population groups of East Asian descent may have a higher prevalence of genetic variants that diminish the effectiveness of codeine as a pain reliever [98]. On the other hand, some African American individuals may have genetic variations that result in faster metabolism of certain pain medications. As a result, they might need higher doses for effective pain relief [99].

Cultural beliefs and practices also have a significant impact on shaping preferences for managing pain [38]. Native American communities often incorporate traditional healing methods such as sweat lodges and herbal remedies to address pain, which are deeply rooted in their cultural beliefs [69]. Similarly, some Hispanic cultures prefer "curanderos" (healers) who combine spiritual rituals with herbal remedies for pain relief [93]. Individuals from these backgrounds may seek out these treatments in addition to or instead of conventional medical care.

Socioeconomic factors also contribute to disparities in the treatment of pain [100]. Urban African American populations with low incomes may face challenges in accessing healthcare, leading to delays in the diagnosis and treatment of pain [77]. Similarly, Hispanic communities residing in rural areas may encounter difficulties in accessing specialized pain clinics, resulting in disparities regarding the availability of advanced pain management options like interventional procedures or multidisciplinary pain teams [101].

Addressing healthcare disparities remains an ongoing concern, as research indicates differences in the treatment of pain based on race and ethnicity [33]. For instance, healthcare providers may be less likely to prescribe opioids for managing pain among African American patients compared to their White counterparts with similar conditions [102]. Furthermore, it's important to consider that language barriers and cultural disparities in expressing pain can contribute to the underestimation of pain severity in Asian American patients. This, in turn, may lead to suboptimal pain management [103].

Trust issues and communication gaps can also have a significant impact on the experience of pain treatment [104]. Historical injustices, including the Tuskegee Syphilis Study, have resulted in long-standing mistrust of the healthcare system among African Americans. Consequently, there may be hesitancy in this community when it comes to discussing pain symptoms or seeking alternative treatments [105]. Similarly, Native American communities may have faced instances of cultural insensitivity within healthcare settings, resulting in strained relationships between patients and providers. Consequently, there might be a reluctance to openly communicate about concerns related to pain [106].

These examples demonstrate how a combination of genetic factors, cultural disparities, socioeconomic conditions, healthcare system dynamics, communication barriers, and trust issues can lead to variations in experiences with pain treatment across different racial and ethnic groups [107]. Healthcare providers should be mindful of these differences and strive towards providing fair, culturally sensitive, and personalized approaches to pain management for all patients regardless of their racial or ethnic background [47]. Promoting equality in pain treatment outcomes for all individuals requires addressing healthcare disparities and fostering trust and open communication [108].

In our concluding thoughts and future perspective, it is crucial to follow evidence-based guidelines for effective and safe pain management [109]. An accurate assessment and diagnosis play a vital role in understanding the cause, severity, and impact of pain [110]. Utilizing validated pain assessment tools helps quantify pain intensity and monitor changes over time [78].

An evidence-based approach often recommends a multidisciplinary approach to managing chronic pain conditions. This encompasses medical, physical, psychological, and complementary therapies tailored to meet each patient's unique needs with the goal of holistic pain relief [111].

When it comes to pharmacological interventions, following evidence-based recommendations is key. Non-opioid analgesics like acetaminophen and NSAIDs are typically recommended as first-line treatments for mild to moderate pain [112]. In cases where opioids are necessary for moderate to severe pain, guidelines emphasize cautious use. This involves conducting risk assessments, regular monitoring, and utilizing prescription monitoring programs to mitigate the risks of addiction and overdose [113]. In addition, it may be helpful to consider using other medications like tricyclic antidepressants and anticonvulsants to effectively manage neuropathic pain [114].

Non-pharmacological approaches are an important part of evidence-based pain management [115]. These include physical therapy and exercise programs to improve physical function [116], cognitive behavioural therapy, and mindfulness-based interventions to address the emotional and psychological aspects of chronic pain [117], interventional procedures such as nerve blocks or epidural injections when necessary [47], and complementary therapies like acupuncture or massage for targeted pain relief [118].

Educating patients is crucial in following an evidence-based approach, providing them with information about their condition, treatment options, and strategies for self-management [119]. Regular assessment and monitoring of pain levels and treatment effectiveness help make adjustments based on the patient's response and any changes in their pain condition [93]. Involving patients in shared decision-making respects their preferences and values regarding their care [120]. Lastly, risk assessment and strategies to minimize risks are particularly important in pain management. Guidelines emphasize thorough assessment and monitoring when prescribing opioids for long-term use [121]. It's crucial to acknowledge that evidence-based guidelines develop over time as new research emerges. This means that healthcare providers need to stay informed about the latest recommendations to provide the most effective care [64]. Furthermore, treatment plans should be tailored to meet each patient's individual needs and the specific characteristics of their pain condition. This ensures that the best possible outcomes are achieved in pain management [122].

Clinical acumen differences when treating pain amongst different ethnicities

Significant connections emerge when considering the intricate interplay among ethnicity, genetic variations, and cultural attitudes in shaping pain perception and treatment [123]. The landscape of pain sensitivity is nuanced by genetic factors, particularly mutations in the SCN9A gene prevalent among South Asian populations [124]. The SCN9A gene, responsible for encoding the Nav1.7 sodium channel, a key player in pain signalling, underscores the importance of comprehending genetic variations for precise pain management [123]. For individuals of South Asian descent, these genetic insights offer potential avenues for tailoring medication prescriptions targeting sodium channels, optimizing the effectiveness of pain management strategies [124].

Cultural attitudes significantly contribute to diverse pain experiences across different ethnic groups [125]. In Hispanic cultures, pain is deeply embedded as an intrinsic part of life, influenced by strong family bonds and cultural values [126]. Healthcare providers engaging with Hispanic patients must navigate these cultural nuances to gain a comprehensive understanding of the patient's pain experience [125]. Culturally sensitive pain management strategies not only acknowledge but also respect and align with these cultural perspectives, fostering more empathetic and effective care [127].

Similarly, linguistic nuances in articulating pain within African American communities highlight the importance of recognizing diverse expressions and descriptions related to pain [128]. Healthcare providers need to be attuned to these linguistic variations to accurately assess and communicate about pain in African American patients [129]. This linguistic awareness becomes crucial in developing personalized and effective pain management plans, ensuring transparent communication between healthcare providers and patients [130].

Beyond genetic and cultural dimensions, psychosocial factors further shape the ethnicity-pain relationship [129]. Studies indicate that cultural beliefs and social norms significantly impact how individuals interpret and respond to pain [126]. The perception of pain as a natural part of life or as a sign of strength varies across cultures [124]. Language, with its unique vocabularies and expressions for describing pain, adds another layer to these cultural differences, influencing how individuals communicate their pain experiences [121]. For example, there is a direct correlation between an increased use of metaphorical language in the extent to which pain interferes with one's life [121]. 

Moreover, disparities in pain management experiences among different racial backgrounds are linked to conscious or unconscious biases held by healthcare providers [123]. These biases, often based on unfounded beliefs about minority populations, contribute to variations in pain treatment approaches [127]. In primary healthcare settings, Black and Hispanic American patients may face additional screening for potential drug abuse compared to White individuals [128]. The intersectionality of race, ethnicity, and biases necessitates a critical examination of healthcare practices.

## Conclusions

In conclusion, the management of pain is a multifaceted endeavor influenced by various sociocultural, demographic, and ethical factors. This review has shed light on the complex interplay of these dimensions in shaping individuals' experiences of pain and their responses to treatment. From sociocultural influences such as cultural beliefs and societal norms to demographic factors like age-related and socioeconomic disparities, each aspect contributes significantly to how pain is perceived and managed.

Furthermore, gender biases, healthcare provider attitudes, and ethical considerations surrounding opioid use add layers of complexity to pain management practices. The evolving landscape of placebo trials underscores the importance of understanding psychological and cultural factors in treatment outcomes.

To address these challenges, evidence-based guidelines, multidisciplinary approaches, and personalized interventions are essential. By acknowledging and addressing the diverse influences on pain experiences, clinicians can provide more effective and tailored care, dismantling systemic barriers, and contributing to the improvement of individual and public health outcomes. Ultimately, advancing pain management practices is crucial for enhancing overall well-being and quality of life for patients suffering from pain.
